# Retrieval of Genuine Ultraviolet Liquid-Microjet Photoelectron Spectra

**DOI:** 10.1021/acs.jpca.5c02024

**Published:** 2025-06-05

**Authors:** Edoardo Simonetti, Helen H. Fielding

**Affiliations:** Department of Chemistry, 4919 University College London, WC1H 0AJ London, U.K.

## Abstract

Ultraviolet liquid-microjet photoelectron spectroscopy is a powerful technique for the determination of electron binding energies of molecules in aqueous solution and for exploring their photochemical dynamics. However, our poor understanding of inelastic scattering of low energy electrons (<10 eV) in water has hindered the determination of accurate vertical ionization energies; although several algorithms have been implemented to retrieve genuine binding energies from experimental spectra, a consensus on the parameters employed is yet to be reached. Here, we investigate the effect of these parameters on the retrieval of true photoelectron spectra of water, phenol, and phenolate. We show that the scattering cross sections, obtained by extrapolating the cross sections in amorphous ice to zero electron kinetic energy, describe the distortion observed in our spectra accurately and that the description of the transmission of electrons at the liquid–vacuum interface is crucial to infer a value for the electron affinity of water at the surface, and we emphasize the importance of considering concentration depth profiles when retrieving true photoelectron spectra of surface-active solutes. Our work highlights the potential for accurate ultraviolet photoelectron spectroscopy of aqueous solutions of organic molecules.

## Graphical abstract

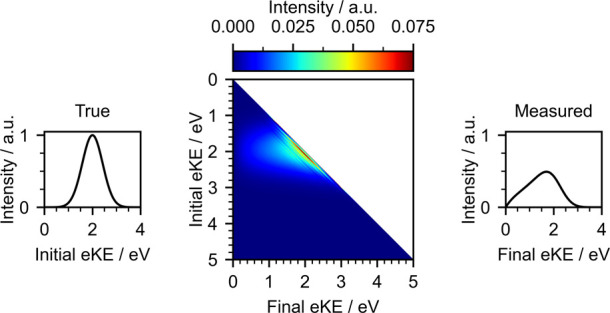

## Introduction

Liquid-microjet photoelectron spectroscopy (LJ-PES) is an indispensable tool for the study of electronic structure and excited state dynamics of solutes in aqueous environments as it enables direct measurement of electron binding energies (eBEs) in solution.^1–6^ However, a huge obstacle for LJ-PES with ultraviolet (UV) light lies in the behavior of low-energy electrons in water as there is not yet a consensus on theoretical descriptions and empirical data for electron scattering in water. It is also one of the greatest challenges hampering our understanding of the effects of ionizing radiation in biology, atmospheric science and nuclear energy.

LJ photoelectron spectra recorded with X-ray and extreme UV (EUV) light sources have the advantage that the distribution of inelastically scattered photoelectrons becomes separated from the distribution of unscattered or elastically scattered photoelectrons because the inelastic scattering events involve energy losses of several eVs.^7^ However, high solute concentrations are needed to obtain sufficiently high signal-to-noise ratios as the photoelectron signal of water (55.5 M) dominates. This has hampered studies of many organic molecules that are only weakly soluble in water (<1 mM). A solution to this problem is to use multiphoton ionization (MPI) with UV laser pulses for the determination of vertical ionization energies (VIEs).^3,8^ Femtosecond time-resolved photoelectron spectroscopy (TRPES) employing a UV pump pulse and a UV or EUV probe pulse, which has proved invaluable for tracking ultrafast electronic dynamics in the gas phase and on surfaces, has also been extended to liquids by several groups.^9–14^ TRPES with EUV probe pulses has the advantage that the observation window is broad enough to track electronic relaxation from the excited-state potential energy surface back to the ground electronic state, but TRPES of sparingly soluble organic molecules requires the increased sensitivity that is best achieved using UV probe pulses. Therefore, UV LJ-PES has the potential to transform our understanding of the electronic structure and relaxation dynamics of photoexcited organic molecules in aqueous solution; however, scattering of the emitted electrons within liquid water is a major challenge that must be overcome for accurate interpretation of these photoelectron spectra.

Recent efforts have attempted to tackle this problem by devising retrieval methods to obtain accurate eBEs from experimental UV photoelectron spectra.^15–19^ The first method, developed by Signorell and co-workers, was based on a Monte Carlo electron transport model to simulate the scattering of low-energy electrons in water employing a set of scattering cross sections derived from amorphous ice and photoelectron spectra of water droplets.^15,20^ Subsequently, Wörner and co-workers simulated spectra with electron kinetic energies (eKEs) between 0 and 50 eV and investigated the effect of different simulation parameters, focusing on photoelectrons generated with EUV pulses.^17^ A different approach involving a spectral inversion method based on empirical results was developed by Suzuki and co-workers.^16,19^ They measured photoelectron spectra of the solvated electron employing EUV light and a range of UV wavelengths. Each measured UV spectrum, *g*_*k*_(*E*), was mapped to an initial Gaussian distribution, *G*_*k*_(*E*), created by shifting the EUV spectrum by the difference between the UV and the EUV photon energies. To obtain true photoelectron spectra, a linear combination of *g*_*k*_(*E*) distributions was fit to experimental data, *I*_meas_ = ∑_*i*_ *c*_*i*_ *g*_*k*_(*E*), and the expansion coefficients, *c*_*i*_, were used to expand the *G*_*k*_(*E*) distributions to obtain true photoelectron spectra, *I*_true_ = ∑_*i*_ *c*_*i*_ *G*_*k*_(*E*). We then combined the spectral inversion approach with Monte Carlo simulations to determine the linear transformations between genuine and measured distributions, and introduced the ability to treat nonuniform concentration depth profiles.^18^ In this approach, each *G*_*k*_(*E*) corresponds to a single photoionization process and the transformation from *G*_*k*_(*E*) to *g*_*k*_(*E*) was obtained from Monte Carlo simulations of electrons in water.

There has been some discussion in the literature over the parameters employed in Monte Carlo simulations and spectral retrieval methods. Signorell and co-workers derived a set of scattering cross sections by using the scattering cross sections measured in amorphous ice as the initial guess to fit to water droplet photoelectron imaging data,^15,20^ which we also employed in our previous work.^18^ In a later study, Signorell refined the set of cross sections they employed by refitting to the droplet data using additional sampling points,^21^ which reduced the value of the cross sections for eKEs below 1 eV. Wörner and co-workers employed a dielectric model of water to determine singly differential inelastic cross sections and scaled the total integral inelastic cross sections to correspond to a constant 3 nm inelastic mean free path.^17^

Another important aspect is the treatment of the transmission of electrons at the surface of liquid jets. Signorell’s group determined the escape for each electron individually,^15^ while Wörner’s group and our group applied a transmission function a posteriori, assuming a uniform distribution of escape angles.^17,18^ Suzuki and co-workers fit this transmission function to the lower energy side of photoelectron spectra and obtained a value of 0.2 eV for the escape threshold.^19^ This value contrasts with those employed by Signorell’s group and our group (1.0 eV),^15,18^ Wörner’s group (0.8 eV)^17^ and a value determined computationally (0.8 eV),^22^ but is consistent with the value suggested by Bartels (0.1 eV).^23^

Inspired by this general lack of consensus, we have undertaken a systematic study of the impact of different parameters on the retrieval of UV photoelectron spectra using a refined version of our earlier spectral retrieval method. We tested the effect of several sets of cross sections, the way the transmission at the liquid–vacuum interface is determined and the value of the escape threshold on the photoelectron spectrum of water. We also revised the depth profiles employed to describe the photoelectron spectra of aqueous phenol and phenolate, which are being used by several groups to benchmark accurate procedures.^8,18,24–28^

## Methods

### Monte Carlo Simulation and Basis Functions

The Monte Carlo simulation describes the transport of electrons inside the liquid jet and their transmission at the liquid–vacuum interface. Electrons are initialized uniformly inside a cylinder with a set radius and infinite height. Several random walks (typically 10^4^) are performed at each starting depth *d*, and initial kinetic energy *E*_i_, which is expressed relative to the vacuum level around the liquid jet. In each step, the electron travels in a random isotropic direction, covering a distance sampled from an exponential distribution with the mean free path (MFP) of the electron as its mean. The MFP is determined using a set of integral cross sections as follows

$$\mathrm{MFP}(E)=\frac{1}{\rho_{n}\sigma_{\mathrm{tot}}(E)}$$ 1

where ρ_*n*_ is the number density of water and σ_tot_(*E*) is the sum of all the integral scattering cross sections at a specific eKE. If the electron is inside the jet, an energy-loss channel describing a translational, rotational or vibrational mode of water is selected based on the relative intensity of the corresponding cross section and the electron loses energy sampled from a normal distribution using parameters obtained from amorphous ice experiments (Table S1 in the Supporting Information).^29^ If at the end of a step the position of the electron places it outside the jet, it must be determined whether the electron has enough energy to escape. Classically, the component of its velocity perpendicular to the surface must correspond to a kinetic energy higher than the electron affinity of water at the surface (*E*_0_), i.e., the difference between the conduction band minimum at the liquid–vacuum interface and the vacuum level. Two approaches have been employed to describe transmission at the surface. The first approach assumes that the distribution of the velocity vectors of electrons hitting the surface is isotropic and uses the transmission probability $T(E)=1-\sqrt{E_{0}/(E+E_{0})}$ to filter electrons at the end of their trajectory (method **A**).^16,29^ An alternative approach is to calculate the kinetic energy component perpendicular to the surface for every electron as it hits the surface and to allow it to escape only if this value is greater than *E*_0_ (method **B**).^15^ Trajectories are terminated when electrons successfully escape the jet or their kinetic energy becomes negative.

The final kinetic energies *E*_f_ are binned in 0.01 eV steps to form a series of distributions *S*(*E*_f_;*E*_i_, *d*), an example of which is shown in Figure 1a. They can be scaled by any arbitrary concentration profile and integrated over starting depth *d*, to give solute-specific basis sets composed of *S*(*E*_f_;*E*_i_) functions. A set employing a uniform concentration profile is shown in Figure 1b. In our previous work, each *S*(*E*_f_;*E*_i_) distribution was normalized by its area in an attempt to create a probability distribution for electrons with a specific initial kinetic energy. However, this fails to account for the fact that electrons with different *E*_i_ do not have the same probability of escaping the jet. In our revised version we retain the relative intensities of the *S*(*E*_f_;*E*_i_) distributions. The transformation from *G*_*k*_(*E*) to *g*_*k*_(*E*) is obtained by scaling the basis set by *G*_*k*_(*E*) and integrating over *E*_i_ (Figure 1c).

**Figure 1:**
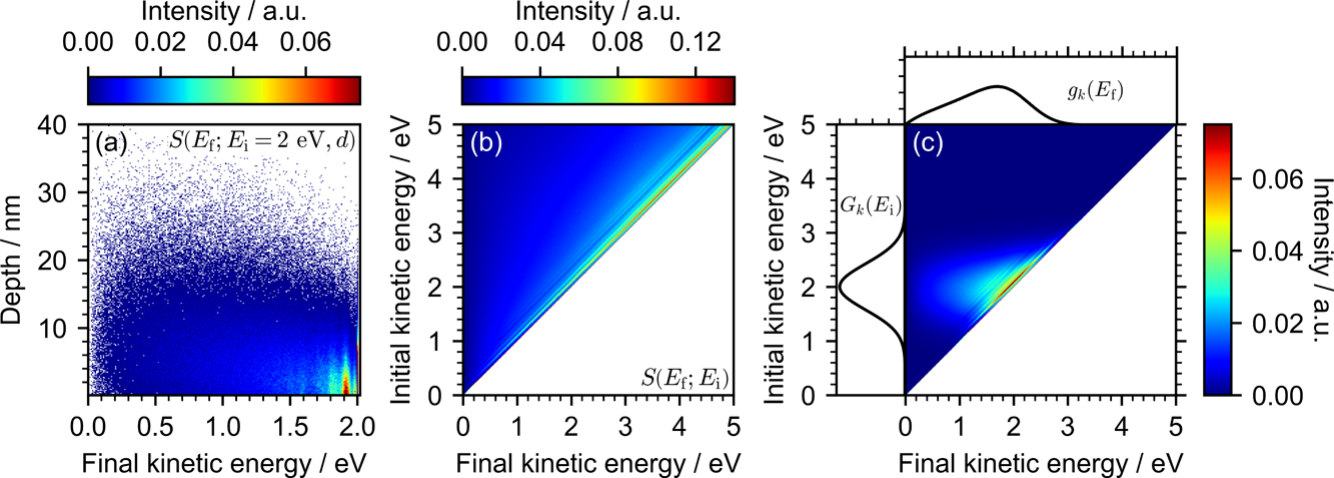
(a) Example normalized *S*(*E*_f_;*E*_i_ = 2 eV, *d*) distributions. Points with intensities between 0.075 and 1 only appear at 2 eV and represent electrons that have only been elastically scattered; this whole range was set to dark red to highlight the variation in the distribution of inelastically scattered electrons. (b) Basis set composed of a series of *S*(*E*_f_;*E*_i_) distributions representing a uniform concentration profile. Points with intensities between 0.14 and 1 only appear on the diagonal and represent electrons that have only been elastically scattered; this whole range was set to dark red to highlight the variation in the distributions of inelastically scattered electrons. (c) Transformation of a true Gaussian into a distorted Gaussian: *G*_*k*_(*E*) ⇒ *g*_*k*_(*E*). The basis set in (b) was scaled by *G*_*k*_(*E*) and integrated across *E*_i_ to generate *g*_*k*_(*E*). In this simulation, we employed starting energies between 0.01 and 5 eV sampled in 0.01 eV steps, depths between 0.1 and 50 nm sampled in 0.1 nm steps, 10 000 electrons at each depth, an escape threshold of 1.0 eV, method **B** to treat the transmission at the surface and cross section set **III**.

### Input

In this work, we simulated electrons in a cylinder with a diameter of 20 μm. We employed starting eKEs between 0 and 5 eV in 0.01 eV steps, the range appropriate for most UV photoelectron spectra, and chose probing depths spanning the jet from the surface to 50 nm below the surface in 0.1 nm steps, as we observed a negligible number of electrons successfully escaping the jet from larger depths (Figure S10 in the Supporting Information). The number of electrons initialized at each depth and initial kinetic energy was chosen to be 10,000, equal to 2.5 × 10^9^ total trajectories. The effects of varying the above parameters are shown in Section S6 in the Supporting Information.

In the absence of experimental data for liquid water, we employed the scattering channels and energy-loss parameters determined for amorphous ice^29^ (Table S1 in the Supporting Information). We tested the effect of four different sets of cross sections (Figure 2). Set **I** employed cross sections digitized from the first spectral retrieval model reported by Signorell’s group based on amorphous ice cross sections and cross sections obtained by fitting to photoelectron spectra of water aerosol droplets.^15,20^ We note that the contribution from the ‘other’ scattering channel is missing from set I below 3 eV because the cross sections in ref 15 were only presented above 2 × 10^−19^ cm^2^; this does not impact the retrieval, as explained in Section S1.1 of the Supporting Information. This set allowed for comparison with our first spectral retrieval model.^18^ Set **II** employed total inelastic and elastic cross sections refined with additional sampling points, as reported by Signorell.^21^ We interpolated these with a cubic spline, and determined the contributions of individual inelastic cross sections relative to the total inelastic cross sections by scaling the amorphous ice cross sections.^29^ Set **III** employed the cross sections for amorphous ice^29^ that we extrapolated linearly to 0 eV below the lowest reported value of 1.7 eV, and interpolated with a cubic spline above 1.7 eV. Set **IV** employed total inelastic and elastic cross sections reported by Signorell’s group and derived by fitting photoelectron spectra of water droplets,^20^ together with the cross sections for amorphous ice above 5.2 eV.^29^ We fit a straight line through the total inelastic cross sections from 3 to 1 eV, extrapolated the line to 0 eV, and interpolated with a cubic spline above 3 eV. We linearly extrapolated the elastic cross sections below 1 eV, and interpolated with a cubic spline above 1 eV. The contributions of individual inelastic cross sections relative to the total inelastic cross sections were determined by scaling the amorphous ice cross sections.^29^

**Figure 2:**
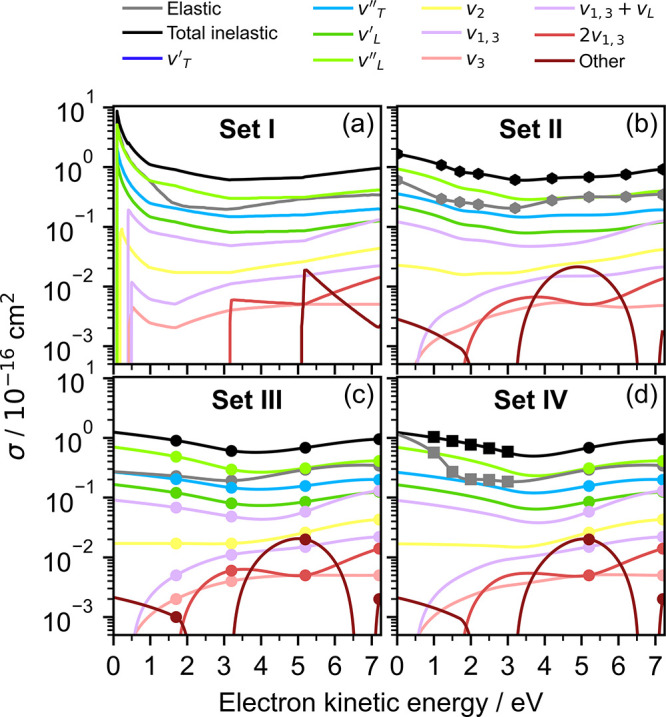
Four sets of cross sections employed in this work, for specified energy loss channels as a function of electron kinetic energy. (a) Set **I**: cross sections digitized from ref 15. (b) Set **II**: cross sections derived from the refined total cross sections in ref 21 (hexagons). (c) Set **III**: cross sections interpolated and extrapolated using the cross sections determined in amorphous ice experiments (circles).^29^ (d) Set **IV**: cross sections determined using photoelectron spectroscopy experiments of water nanodroplets (squares)^20^ and amorphous ice experiments (circles).^29^

We employed a uniform concentration depth profile for water and distributions formed from a Gaussian centered near the surface and a vertical offset to represent phenol and phenolate, guided by molecular dynamics (MD) simulations at the liquid–vacuum interface (Section S3 in the Supporting Information).

## Results and Discussion

### Scattering Cross Sections

Figure 3 shows the photoelectron spectrum of water following nonresonant two-photon ionization with a 200.2 nm pulse^18^ fit with (a) a Gaussian, or (b–e) our method using different sets of scattering cross sections. The experimental data were corrected to account for the reduced collection efficiency at low eKE, streaming potential and vacuum-level offset, and reflect the distribution of photoelectrons emitted from the surface of the jet. Simulations were run using an escape threshold of 1.0 eV and method **B** was employed to determine the transmission of electrons at the surface. The results of the fits are summarized in Table 1. Fitting the spectrum with a Gaussian neglects inelastic scattering and incorrectly overestimates the VIE of water as 11.51 ± 0.07 eV, which is 0.2 eV higher than the accurate values determined using X-ray PES by Kurahashi et al. (11.31 ± 0.04 eV)^30^ and Thürmer et al. (11.33 ± 0.03 eV).^31^ Employing cross section set **I**, which was introduced by Signorell and co-workers^15^ and employed in our previous retrieval code,^18^ gave a negative central eKE corresponding to a VIE of 12.49 ± 0.09 eV. A negative value was obtained because low energy electrons are underrepresented by this basis set, thus a large true signal at very low eKE is needed to account for the experimentally observed signal. This indicates that the cross sections at eKEs less than around 1 eV are too high. In their revised set of cross sections, Signorell reduced the contribution of the cross sections below 1 eV;^21^ using cross section set **II** resulted in a VIE of 11.43 ± 0.09 eV, which is much closer to the X-ray values.^30,31^ Finally, we employed the two sets of cross sections derived from the extrapolation of amorphous ice cross sections^29^ (**III**) and the combined amorphous ice and water droplet photoelectron data^20^ (**IV**) and obtained VIEs of 11.31 ± 0.09 eV and 11.36 ± 0.09 eV, respectively. Both values are consistent with the VIE of water determined using X-ray PES.^30,31^ In fact, the only notable difference between the two cross section sets lies in the magnitude of the elastic cross sections, as the total and most intense inelastic cross sections are not affected greatly. As expected, this has a smaller effect on the central eKE of the retrieved spectra. Motivated by these results, we employed the extrapolated amorphous ice cross section set **III** in the following sections, as it provides good agreement with the literature.

**Figure 3:**
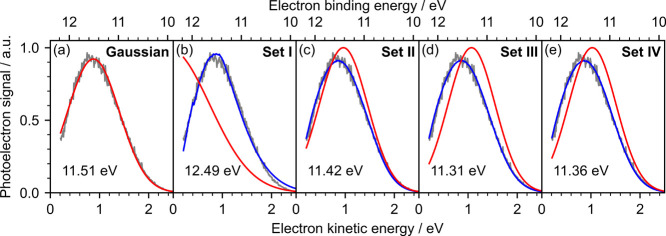
Photoelectron spectrum of water recorded at 200.2 nm as a function of electron kinetic energy interpolated in 0.01 eV steps. The spectrum was fitted with a Gaussian (a) or fitted using our spectral retrieval code using cross section sets **I–** **IV** (b–e). Gray lines are the experimental spectra, blue lines are fits to the data, red lines are retrieved spectra, and numbers in eV are the ionization energies of water determined for each method. All spectral retrievals employed method **B** with an escape threshold of 1.0 eV. Experimental spectrum reproduced from ref 18. Copyright 2022 American Chemical Society.

**Table 1:** Central eKEs, eBEs, and FWHMs of the 200.2 nm Spectrum of Water^18^ Obtained by Fitting a Gaussian or with Our Retrieval Code Employing Cross-Section Sets **I–IV**

method	eKE/eV	eBE/eV	FWHM/eV
Gaussian	0.88 ± 0.07	11.51 ± 0.07	1.21 ± 0.04
set **I**	–0.11 ± 0.09	12.49 ± 0.09	2.00 ± 0.09
set **II**	0.96 ± 0.09	11.43 ± 0.09	1.17 ± 0.07
set **III**	1.08 ± 0.09	11.31 ± 0.09	1.13 ± 0.07
set **IV**	1.02 ± 0.09	11.36 ± 0.09	1.14 ± 0.07

### Escape Threshold

Figure 4a shows the distribution of the angles of incidence on the surface for electrons with kinetic energies between 0 and 5 eV that successfully escape the jet. Here, method **B** was employed, rather than assuming a uniform distribution of the velocity vectors of electrons reaching the surface. If the latter and no escape barrier are assumed, one would expect a sinθ distribution of the incident angles.^16,19,29^ However, our results show that the distribution more closely resembles a sin­(2θ) distribution and has a maximum slightly below π/4 radians. Assuming electrons are uniformly distributed at all depths below the surface and the distance between two consecutive scattering events follows an exponential distribution, we show in Section S5 in the Supporting Information that the distribution of incidence angles follows a sinθ distribution only if electrons escape from depths much smaller than the MFP.^32^ This assumption is valid for films composed of a few monolayers of molecules and was employed in modeling electron scattering in films of amorphous ice.^29^ However, since the radius of a liquid jet is much larger than the MFP of electrons in water, greater depths must be accounted for and a sin­(2θ) distribution is obtained (eq S8 in the Supporting Information).

**Figure 4:**
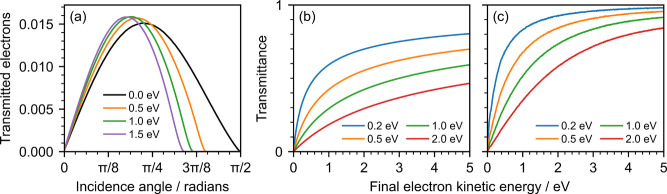
(a) Fraction of simulated electrons successfully escaping the jet as a function of the incidence angle with the surface, for different escape thresholds, determined using method **B**. (b, c) Ratio between the number of transmitted electrons in the presence of different escape barriers and the number of transmitted electrons without an escape barrier, as a function of final kinetic energy. Transmission was determined using (b) method **A**, and (c) method **B**. In these simulations, we employed cross section set **III**.

Additionally, we performed simple random walks in which we restricted collisions to elastic isotropic scattering events and sampled the step lengths from an exponential distribution with a constant MFP (Figure S5 in the Supporting Information). We found an escape angle distribution with its maximum less than π/4 and a depth distribution which could be described by a shifted cumulative exponential distribution. We then showed that the distribution of electrons inside the jet affects the position of the maximum of the escape angle distribution (Figure S6 in the Supporting Information). In fact, as electrons approach the surface, they are increasingly likely to escape the jet, thus depleting the population of electrons near the surface. As these electrons have a higher likelihood of escaping the jet at larger angles, the most likely incidence angle shifts to lower values.

For nonzero escape barriers, this effect is more pronounced as transmission does not occur above the maximum escape angle of electrons with an eKE of 5 eV (Figure 4a). Notably, the fraction of electrons transmitted at lower angles is greater when a barrier is present. We attribute this to the fact that electrons that are reflected into the liquid will find themselves close to the surface and are thus likely to escape at a more favorable angle.

Overall, this phenomenon leads to a significantly different kinetic energy distribution of the transmitted electrons than when using method **A**. Figure 4b,c shows the ratio between the number of transmitted electrons in the presence of an escape barrier and the number of transmitted electrons without an escape barrier for both methods. Method **B** results in a larger number of transmitted electrons overall and a more significant contribution from lower energy electrons. We derived analytical functions for the transmittance of electrons at the surface using a uniform distribution and a cumulative exponential distribution and these are presented in Section S5 in the Supporting Information (eqs S12 and S17). This effect can be observed when applying our spectral retrieval to the 200.2 nm photoelectron spectrum of water (Figure 5a,b). Method **A** requires an escape threshold 5 times lower than method **B** to describe the spectrum accurately. This suggests that the discrepancy between the escape threshold obtained by Suzuki’s group (0.2 eV)^19^ and that employed by Signorell and co-workers^15^ and in our previous work (1.0 eV)^18^ can be attributed to the misassumption that the distribution of incidence angles of electrons at the surface is sinθ.

**Figure 5:**
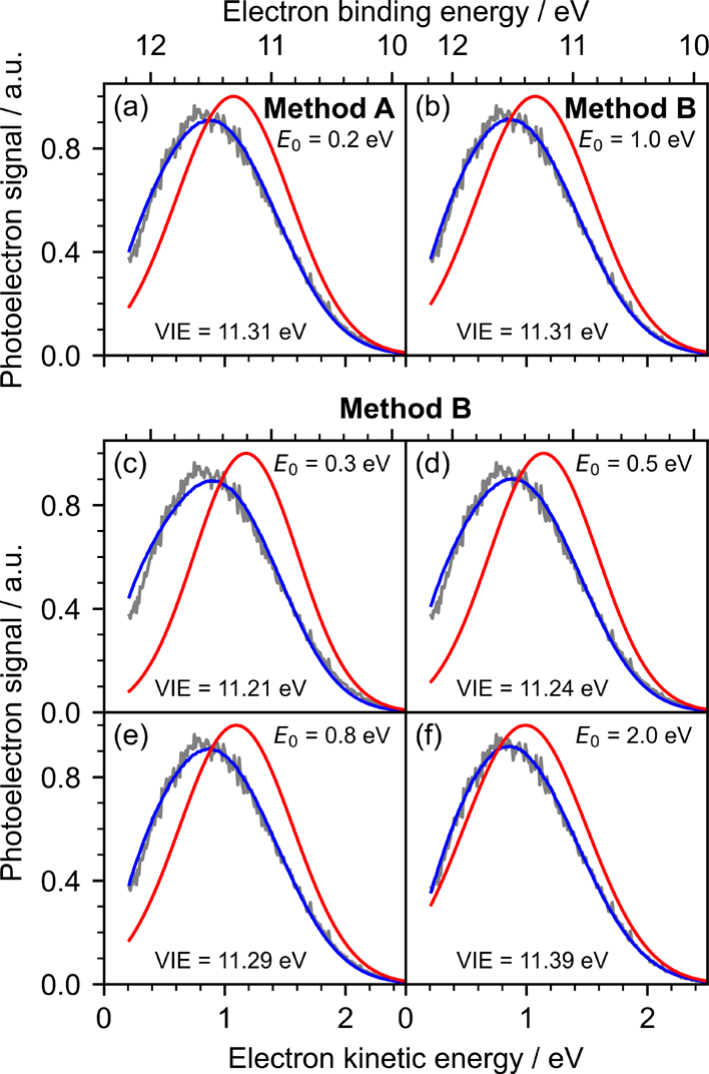
Photoelectron spectrum of water recorded at 200.2 nm retrieved using the two methods of calculating the transmission of electrons at the liquid–vacuum interface with different escape thresholds. Gray lines are the experimental spectra, blue lines are fits to the data, red lines are retrieved spectra, and numbers in eV are the ionization energies of water determined for each method. (a) Method **A** with an escape threshold of 0.2 eV was used. (b) Method **B** with an escape threshold of 1.0 eV was used. (c–f) Method **B** with escape thresholds between 0.3 and 2.0 eV was used. All spectral retrievals employed cross section set **III**. Experimental spectrum reproduced from ref 18. Copyright 2022 American Chemical Society.

Next, we investigated the effect of the value of the escape threshold on the retrieval of the 200.2 nm photoelectron spectrum of water using method **B** (Figure 5c,d). As the spectrum has a significant contribution from electrons with eKEs below 1 eV, the choice of escape threshold affects the position of the retrieved peak significantly, and higher *E*_0_ values yield better fits and values more consistent with previous accurate measurements.^30,31^ It is worth noting that the shape of distorted spectra at low eKE is also dictated by the scattering cross sections at these eKEs and more than one combination of cross section set and escape threshold yield the same result. We opted for an escape threshold of 1 eV and employed it with cross section set **III** to retrieve the spectra of water, phenol and phenolate, because this value was consistent with previous studies and yields VIEs and VDEs consistent with X-ray PES experiments.

### Concentration Depth Profile

Figure 6a shows the distribution of phenol and phenolate as a function of depth below the surface, determined using molecular dynamics simulations. Both molecules have an enhanced surface concentration and a bulk contribution to the concentration depth profile. The ratio between the surface and bulk contributions is consistent with surface tension and photoelectron spectroscopy studies of phenol and phenolate, which showed a stronger surface propensity for phenol.^27,28^ Since our simulation does not model the fall in the density of water at the interface, we approximated the depth dependence of the concentration as the sum of a Gaussian centered 0.1 nm below the surface with a 0.4 nm FWHM and a vertical offset (Figure 6a), and obtained ratios between the height of the Gaussian and the offset of 20 and 1 for phenol and phenolate, respectively.

**Figure 6:**
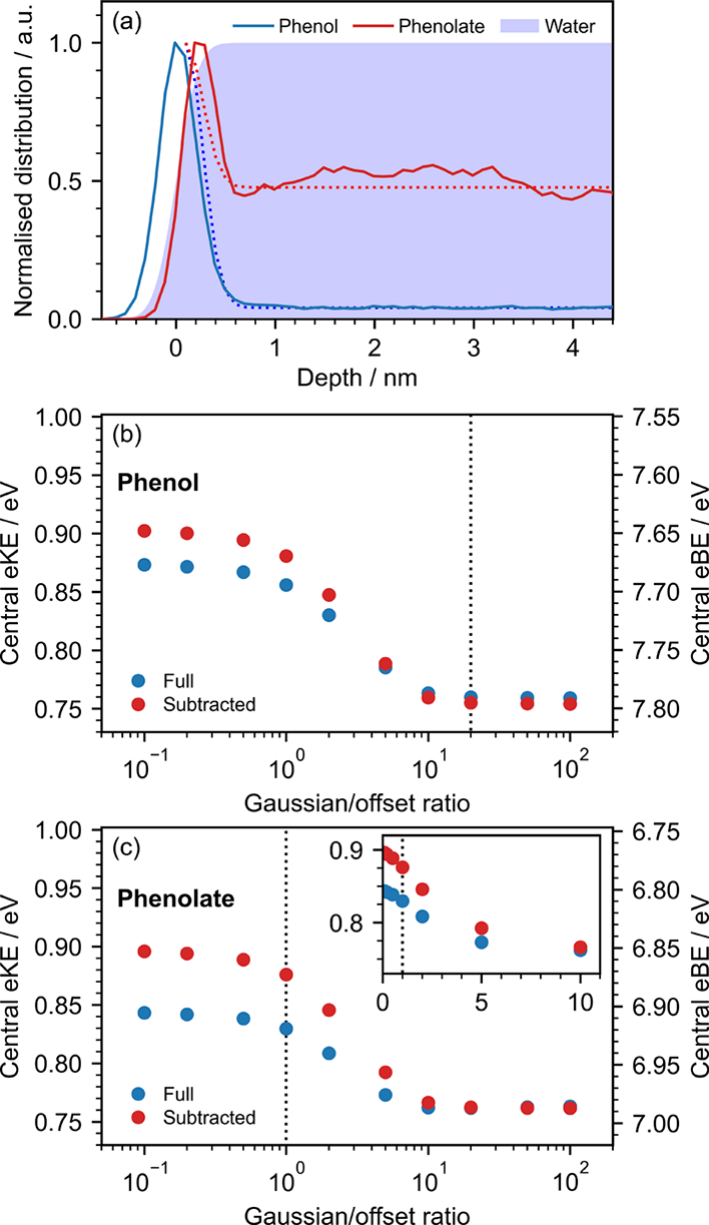
(a) Normalized concentration depth profile of phenol (blue) and phenolate (red) in aqueous solution near the liquid-vacuum interface, determined from molecular dynamics simulations. The center of mass of each solute molecule was used to track its position along the axis perpendicular to the surface. The blue shaded area represents the distribution of the oxygen atoms of water molecules fit with a cumulative normal distribution normalized to 1; its mean is taken as the liquid–vacuum interface. Dotted lines represent the concentration profiles employed in the retrieval of the photoelectron spectra of phenol and phenolate. (b) Retrieved electron kinetic and binding energies of the 290 nm two-photon nonresonant MPI phenol spectrum including (blue) and excluding (red) solvent contributions as a function of relative height of the surface Gaussian. (c) Retrieved electron kinetic and binding energies of the 320 nm two-photon nonresonant MPI phenolate spectrum including (blue) and excluding (red) solvent contributions as a function of relative height of the surface Gaussian. Inset: retrieved electron kinetic energies plotted on a linear scale. Vertical dotted lines indicate the ratios obtained from (a).

Figure 6b,c shows the dependence of the retrieved eBEs of phenol and phenolate on the ratio between the height of the Gaussian and the offset. Here, we employed our retrieval process for the nonresonant 1 + 1 MPI photoelectron spectra of 0.1 mM solutions of phenol and phenolate.^18^ For both molecules, the retrieved eKE decreases rapidly with the height of the Gaussian and reaches a plateau at around 10, resulting in a shift of up to around 0.15 eV between a purely bulk and a purely surface species. Vertical dotted lines indicate the Gaussian-offset ratio observed in our MD simulations.

Figure 7a–d shows the retrieval of the nonresonant photoelectron spectra of 0.1 mM solutions of phenol and phenolate, obtained with Gaussian-offset ratios of 20 and 1, respectively. The experimental data were corrected to account for the reduced collection efficiency at low eKE, streaming potential and vacuum-level offset, and reflect the distribution of photoelectrons emitted from the surface of the jet. The spectra in Figures 7a,b show contributions from both the solute (pink Gaussians) and the solvent (green Gaussians), while those in Figure 7c,d were obtained by subtracting the fits of solvent-only spectra (Figure S14 in the Supporting Information).

**Figure 7:**
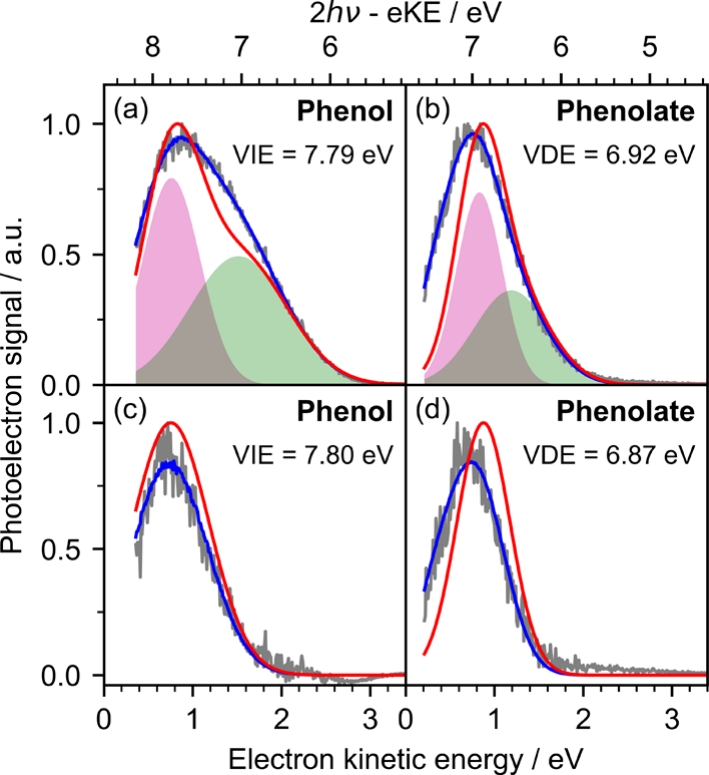
Retrieval of the (a, c) 290 nm photoelectron spectra of 0.1 mM phenol and (b, d) 320 nm photoelectron spectra of 0.1 mM phenolate. Spectra (a) and (b) include solute (pink Gaussians) and solvent (green Gaussians) contributions. Solvent-only spectra were fit with a ‘bulk’ Gaussian (Figure S14 in the Supporting Information) and subtracted from spectra (a) and (b) to give the solute-only spectra in (c) and (d). Gray lines are the experimental spectra, blue lines are fits to the data, and red lines are retrieved spectra. All spectral retrievals employed cross section set **III**, and method **B** with an escape threshold of 1 eV. Experimental spectra reproduced from ref 18. Copyright 2022 American Chemical Society.

The spectrum of 0.1 mM phenol comprises a ‘surface’ Gaussian and a ‘bulk’ Gaussian originating from two-photon nonresonant ionization of phenol and three-photon nonresonant ionization of water (Table 2). VIEs of 7.79 ± 0.09 and 7.80 ± 0.09 eV were obtained for the full and subtracted spectra, respectively. Several studies have reported the VIE of aqueous phenol. An X-ray PES work found a VIE of 7.8 ± 0.1 eV for a 750 mM solution,^24^ which was obtained by calibrating the spectrum using a value for the VIE of water of 11.16 eV.^1^ A more recent study by Yamamoto et al. investigated the VIE of phenol recorded with 40.0 eV femtosecond pulses as a function of phenol concentration.^28^ They observed a decrease in the VIEs of water and phenol at higher phenol concentrations, which they attributed to a decrease in surface potential caused by a higher surface concentration of the solute. For a solution of 25 mM, the measured VIE of water was the same as the VIE of pure water, suggesting that surface potential effects were insignificant and thus they quoted a value of 7.90 ± 0.04 for the VIE of phenol.^28^ Furthermore, a velocity-map photoelectron imaging study of aqueous phenol aerosol droplets also observed a decrease in the VIE of phenol from 7.9 ± 0.1 eV for a 10 mM solution to 7.4 ± 0.1 eV for a 800 mM solution and attributed this trend to the formation of aggregates of phenol at the surface at higher concentrations.^33^ We expect our results to be unaffected by a shift in surface potential or the formation of aggregates, as the surface concentration for a 0.1 mM solution is at least 2 orders of magnitude lower than surface concentrations investigated in previous studies.^27,28,33^ We find good agreement between our retrieved VIE of phenol and previously reported VIEs in liquid jets and aerosol droplets.^24,28,33^

**Table 2:** Vertical Binding Energies of Aqueous Phenol and Phenolate

	phenol		phenolate	
	conc./mM	VIE/eV	conc./mM	VDE/eV
UV LJ-PES full (retrieved)	0.1	7.79 ± 0.09	0.1	6.92 ± 0.09
UV LJ-PES subtracted (retrieved)	0.1	7.80 ± 0.09	0.1	6.87 ± 0.09
X-ray LJ-PES 200 eV^a^	750	7.8 ± 0.1	750	7.1 ± 0.1
EUV LJ-PES 40−56.5 eV^b^	25	7.90 ± 0.04	50	7.3 ± 0.1
UV droplet PES 4.30 eV^c^	10	7.9 ± 0.1		

a Ref 24.

b Ref 28.

c Ref 33.

The spectrum of 0.1 mM phenolate can also be described as the sum of a ‘surface’ Gaussian originating from detachment of phenolate and a ‘bulk’ Gaussian originating from ionization of the solvent. The phenolate contribution has a peak at 6.92 ± 0.09 eV, which agrees well with the value obtained from the subtracted spectrum, 6.87 ± 0.09 eV. The VDE of phenolate measured with X-ray PES was reported to be 7.1 ± 0.1 eV for a 750 mM solution,^24^ which was also determined with a value of 11.16 eV for the VIE of water.^1^ More recently, Yamamoto et al. performed EUV measurements and measured a VDE of 7.3 ± 0.1 eV for a 50 mM solution.^28^ Curiously, our value is 0.3 eV less than the value obtained from EUV measurements and within error of the VDE obtained from an X-ray measurement that was calibrated using a value for the VIE of water that has been subsequently refined. A possible explanation for this difference could be that UV LJ-PES has a greater bulk sensitivity than EUV LJ-PES, and our measurements are able to capture the significant bulk contribution of phenolate. In fact, changing the surface Gaussian and bulk offset in the modeled concentration profile of phenolate yields a VDE closer to values measured with EUV light (Figure 6c). We also note that our experimental conditions differ from those employed in EUV and X-ray experiments; namely, we are using concentrations at least 2 orders of magnitude lower than those that are feasible in EUV and X-ray measurements, and we do not apply a bias to our liquid jet.

## Conclusions

Inspired by the lack of consensus on the parameters required to describe electron transport in liquid water to retrieve accurate photoelectron spectra from UV liquid-microjet spectroscopy measurements, we have undertaken a systematic investigation of the low energy (<5 eV) electron scattering cross sections in water and the description and parameters required to describe transmission through the water–vacuum interface. We found that using the cross sections determined from amorphous ice, linearly extrapolated to zero electron kinetic energy, calculating the component of electron kinetic energy perpendicular to the water–vacuum interface for every electron trajectory, and using an escape threshold of 1 eV, allowed us to retrieve values for the vertical ionization energy of water from UV photoelectron spectroscopy measurements that are in excellent agreement with accurate X-ray photoelectron spectroscopy measurements. We also present values for the vertical ionization and detachment energies determined from photoelectron spectra of sub-mM solutions of phenol and phenolate that are in agreement with earlier X-ray photoelectron spectroscopy measurements and measurements in liquid droplets. We have shown that UV LJ-PES has promise as a spectroscopic tool for studying the electronic structure and photochemical dynamics of bulk aqueous solutions and surface active solutes in aqueous solution. Our code for retrieving true photoelectron spectra from measured UV photoelectron spectra of aqueous solutions LJscatter is written in Julia and Python, and is freely available.^34^

## Supplementary Material



## Notes

Description and discussion of the energy-loss parameters and cross sections employed in this work, example basis functions, details of the molecular dynamics simulations, literature values of the VIE of water, and discussion of the distribution of incidence angles, sensitivity analysis, uncertainties in the spectral retrieval, and solvent-only background photoelectron spectra (PDF)
