# Supplementary material for: Retrieval of Genuine Ultraviolet Liquid-Microjet Photoelectron Spectra
Source: J Phys Chem A. 2025 Jun 5;129(24):5292–9. doi: 10.1021/acs.jpca.5c02024 (PMC12186609; doi:10.1021/acs.jpca.5c02024)
Supplement: Supplementary file 1 [file jp5c02024_si_001.pdf]

Supporting Information: Retrieval of Genuine Ultraviolet Liquid-Microjet Photoelectron Spectra

Edoardo Simonetti and Helen H. Fielding*

Department of Chemistry, University College London, WC1H 0AJ London, UK

E-mail: h.h.fielding@ucl.ac.uk.

Contents

S1 Energy-loss parameters and cross sections **S2**

S1.1 Contribution of 'other' cross sections S2

S1.2 Linear and cubic spline interpolation S3

S1.3 Constant inelastic MFP S4

S2 Basis functions **S5**

S3 Molecular dynamics simulations **S6**

S4 Literature values of the VIE of water **S7**

S5 Distribution of incidence angles **S7**

S6 Sensitivity analysis **S12**

S6.1 Number of starting electrons S13

S6.2 Simulation depth and step size S14

S6.3 Radius of the jet S15

S6.4 Relative contribution of elastic and inelastic cross sections S16

S7 Uncertainties in the spectral retrieval **S18**

S8 Solvent-only background photoelectron spectra **S18**

S1. Energy-loss parameters and cross sections

Our Monte Carlo simulation requires energy-loss parameters and cross sections in order to model the magnitude and likelihood of the energy loss that electrons experience in liquid microjets. Table S1 shows the energy-loss parameters employed in this study, which were obtained from electron-scattering experiments of amorphous ice.[1] The parameters describe normal distributions and are associated with energy-loss channels that describe either an elastic or inelastic scattering event. Inelastic scattering channels are represented by translational, librational, and vibrational modes of water; an additional channel named 'other' is present and includes electron attachment, electronic excitation and ionization processes. The trajectories of electrons that accessed the channel 'other' were terminated in our simulations (see Section S1.1 for a detailed discussion and justification).

Table S1: Mean energy and FWHM of the energy-loss channels employed in this study, taken from electron-scattering measurements in amorphous ice.[1] v'_T and v''_T are translational modes, v'_L and v''_L are librational modes, v_2 is the bending mode, $v_{1,3}$ are the stretching modes, v_3 is the asymmetrical stretching mode, $v_{1,3} + v_L$ is the stretching-librational combination mode and $2(v_{1,3})$ is the overtone of the stretching modes of water. 'Other' includes higher energy channels and 'elastic' represents elastic scattering events, which do not involve any energy loss.

Channel	Mean / meV	FWHM / meV
v'_T	10	1
v''_T	25	24
v'_L	61	30
v''_L	92	40
v_2	204	16
$v_{1,3}$	471	50
v_3	460	5
$v_{1,3} + v_L$	500	40
$2(v_{1,3})$	835	75
other[a]	–	–
elastic	0	0

[a]These trajectories are terminated in our code.

S1.1. Contribution of 'other' cross sections

The scattering channel 'other' includes electron attachment, electronic excitation and ionization processes. The energy loss of these processes was not quantified by Michaud et al. as they focused on energy losses below 1 eV.[1] For electrons within the eKE range employed here (0–5 eV), they suggested that the processes included in 'other' were dom-

inated by electron attachment, followed by either dissociation or autodetachment from high vibrational levels of the electronic ground state of the water anion. The latter produces electrons that could be measured in our experiment. However, as the authors did not quote the energy loss or likelihood of this process and its cross sections are relatively low, we opted to terminate these trajectories, effectively only modelling electron capture. To test whether terminating the trajectories that access this channel is justified, we performed extra simulations employing cross-section set **III** and (a) setting the ‘other’ cross sections to 0, (b) setting the ‘other‘ cross sections to 0 and rescaling the rest of the inelastic cross sections to yield the same total inelastic cross sections as set **III**, and (c) setting the ’other’ energy loss to 1.5 eV with a FWHM of 0.25 eV. The results of these simulations are presented in Figure S2 and demonstrate that the ‘other’ channel does not have any significant effect on the retrieval of the 200.2 nm non-resonant two-photon photoelectron spectrum of water.

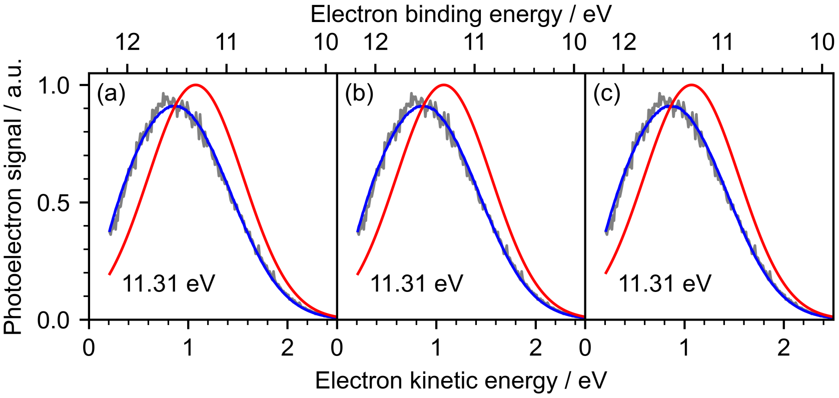

Figure S1: Retrieval of the 200.2 nm non-resonant two-photon photoelectron spectrum of water employing cross-section set **III** and (a) setting the ‘other‘ cross sections to 0, (b) setting the ‘other‘ cross sections to 0 and rescaling the rest of the inelastic cross sections to yield the same total inelastic cross sections as set **III**, and (c) setting the ‘other’ energy loss to 1.5 eV with a FWHM of 0.25 eV. Gray lines are the experimental data, blue lines are fits to the data, red lines are retrieved spectra, and numbers in eV are the ionisation energies of water determined using each method. All simulations were run using method **B** and an escape threshold of 1.0 eV. Experimental spectrum reproduced from ref 2. Copyright 2022 American Chemical Society.

S1.2. Linear and cubic spline interpolation

We employed a cubic spline to interpolate the cross sections employed in our method rather than linear interpolation, to avoid the introduction of artificially sharp features in the distorted distributions (Figure S2c). The retrieval of the spectra employed in this

study is unaffected by the choice of interpolation, as it only has an effect at central eKEs $\gtrsim$ 3 eV (Figure S2 a, b).

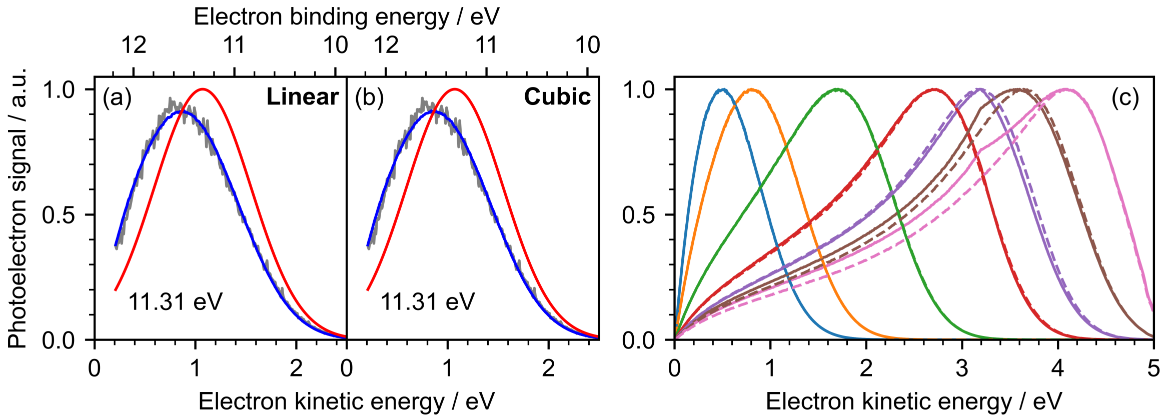

Figure S2: (a b) Effect of interpolating cross-section set **III** (a) linearly or (b) with a cubic spline on the retrieval of the spectrum of water. Gray lines are the experimental data, blue lines are fits to the data, red lines are retrieved spectra, and numbers in each panel are the retrieved eBEs. (c) Effect of interpolating cross-section set **III** linearly (solid lines) or with a cubic spline (dashed lines) on the distortion of Gaussians centred at 0.5, 1, 2, 3, 3.5, 4 and 4.5 eV and a FWHM of 1 eV. All simulations were run using method **B** and an escape threshold of 1.0 eV. Experimental spectrum reproduced from ref 2. Copyright 2022 American Chemical Society.

S1.3. Constant inelastic MFP

Wörner and co-workers employed a constant IMFP of 3 nm in their simulation.[3] Figure S3 shows the retrieval of the spectrum of water employing the amorphous ice inelastic cross sections[1] scaled to give a total IMFP of 3 nm, and the amorphous ice elastic cross sections, which were kept unchanged.

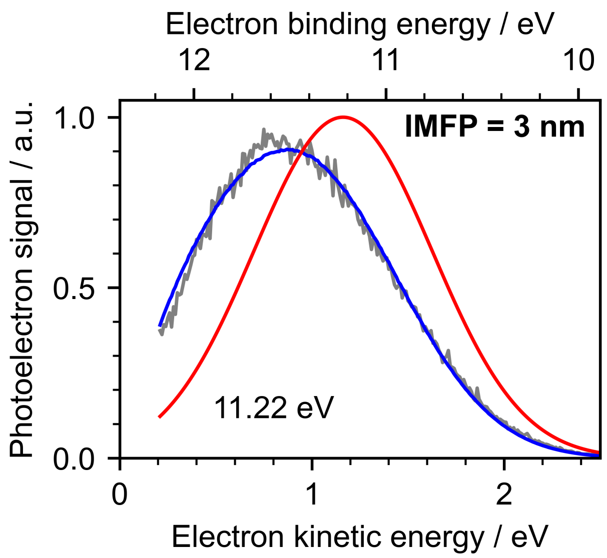

Figure S3: Photoelectron spectrum of water retrieved using cross sections corresponding to an IMFP of 3 nm. The value in eV is the retrieved eBE. The simulation was run using method **B** and an escape threshold of 1.0 eV. Experimental spectrum reproduced from ref 2. Copyright 2022 American Chemical Society.

S2. Basis functions

Figure 1 in the main text shows the contribution of inelastically scattered electrons to $S\left(E_\mathrm{f}; E_\mathrm{i} = 2\ \mathrm{eV}, d\right)$ and $S\left(E_\mathrm{f}; E_\mathrm{i}\right)$ distributions for a uniform concentration profile. The contributions of both elastically and inelastically scattered electrons are presented in Figure S4 for a selection of $S\left(E_\mathrm{f}; E_\mathrm{i}\right)$ distributions.

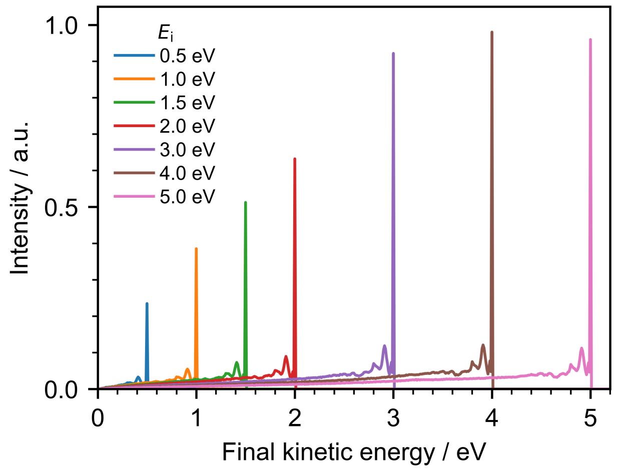

Figure S4: $S\left(E_\mathrm{f}; E_\mathrm{i}\right)$ distributions representing a uniform concentration profile for several E_i values. This simulation was run using method **B** with an escape threshold of 1.0 eV and cross-section set **III**.

S3. Molecular dynamics simulations

We performed molecular dynamics (MD) simulations of phenol and phenolate in aqueous solution, using the OpenMM package (version 7.7.0),[4] to inform our choice of concentration profiles in our retrieval code. The initial coordinates of the solute were optimised using the Automated Topology Builder and Repository[5,6] at the B3LYP/6-31G* level of theory. We employed the SMIRNOFF force field (version 0.11.4)[7] to model the solute surrounded by a Transferable Intermolecular Potential with 3 Points (TIP3P) water environment.[8] One molecule of phenol or sodium phenolate were placed in a 5×5×9 nm box and surrounded with water molecules. The box was then expanded to 5×5×18 nm to simulate the liquid-vacuum interface and periodic boundary conditions were applied. MD simulations were run using Langevin dynamics with LFMiddle discretisation[9] with a heat bath set at 300 K, a 2 fs time step and a friction coefficient of 1 ps^{-1}. Long-range electrostatic interactions were described by the Particle Mesh Ewald (PME) with a 1 nm cutoff. A local energy minimization was performed and the velocities of all the atoms were set to random values chosen from the Boltzmann distribution at 300 K, before the system was allowed to equilibrate for 2 ns. Simulations were run for 120 ns, recording the position of all atoms every 4 ps. Six simulations per solute were carried out. The centre of mass of the solute was used to track its position along the axis perpendicular to the surface.

S4. Literature values of the VIE of water

Table S2: $1b_1$ VIE and FWHM of water from the literature and this work.

Work	Photon energy / eV	VIE / eV	FWHM / eV
Winter et al. (2004)[10]	80–120	11.16 ± 0.04	1.45 ± 0.08
Nishizawa et al. (2011)[11]	516–532	11.23 ± 0.01	1.47 ± 0.01
Kurahashi et al. (2014)[12]	600	11.31 ± 0.04	1.45 ± 0.02
Perry et al. (2020)[13]	21.7–32.5	11.67 ± 0.15	–
Thürmer et al. (2021)[14]	40.813	11.40 ± 0.07	–
Thürmer et al. (2021)[15]	29.97–400.868	11.33 ± 0.03	–
Scholz et al. (2022)[2]	6.20	11.36 ± 0.09	1.27
This work (set **III**)	6.20	11.31 ± 0.09	1.13 ± 0.07

S5. Distribution of incidence angles

In order to understand the origin of the distribution of incidence angles at the liquid-vacuum interface that we obtained in our simulation, we derived the angular distribution of a simplified model in which electrons are uniformly distributed below a flat surface, step lengths are sampled from an exponential distribution with a constant MFP, and all scattering events are elastic and isotropic.[16] The distribution of incidence angles is given by

$$I\left(\theta, d, L\right) = \begin{cases} \frac{1}{2}\sin\theta & 0 \leq \theta \leq \arccos\left(\frac{d}{L}\right) \\ 0 & \arccos\left(\frac{d}{L}\right) < \theta \leq \frac{\pi}{2} \end{cases}. \tag{S1}$$

where θ is the incidence angle, d is the depth and L is the step length. Integration over uniform d and L scaled by an exponential distribution gives the overall angular distribution

$$I\left(\theta\right) = \int_0^{d_{\max}} \int_0^{\infty} I\left(\theta, d, L\right) \frac{1}{\lambda} \mathrm{e}^{-\frac{L}{\lambda}} \,\mathrm{d}L \,\mathrm{d}d, \tag{S2}$$

where $d_{\max}$ is the maximum depth and λ is the mean free path. As $L \geq \frac{d}{\cos\theta}$,

$$I\left(\theta\right)=\int_{0}^{d_{\max}}\int_{d/\cos\theta}^{\infty}\frac{1}{2\lambda}\mathrm{e}^{-\frac{L}{\lambda}}\sin\theta\,\mathrm{d}L\,\mathrm{d}d. \tag{S3}$$

Integrating over L gives

$$I\left(\theta\right)=\frac{1}{2}\sin\theta\int_{0}^{d_{\max}}\mathrm{e}^{-\frac{d}{\lambda\cos\theta}}\,\mathrm{d}d. \tag{S4}$$

Integrating over d gives

$$I\left(\theta\right)=\frac{\lambda}{2}\cos\theta\sin\theta\left(1-\mathrm{e}^{-\frac{d_{\max}}{\lambda\cos\theta}}\right). \tag{S5}$$

If $d_{\max}\ll\lambda$, as can be assumed for the amorphous ice films employed by Michaud et al.,[1] $1-\mathrm{e}^{-\frac{d_{\max}}{\lambda\cos\theta}}\approx\frac{d_{\max}}{\lambda\cos\theta}$ and $I(\theta)$ is given by

$$I\left(\theta\right)=\frac{d_{\max}}{2}\sin\theta, \tag{S6}$$

which can be used to derive the following equation for the transmittance of electrons at the surface:[1,17,18]

$$T(E)=1-\sqrt{\frac{E_0}{E+E_0}}. \tag{S7}$$

However, in a liquid-jet, $d_{\max}\gg\lambda$, and $1-\mathrm{e}^{-\frac{d_{\max}}{\lambda\cos\theta}}\approx1$. $I(\theta)$ is thus given by

$$I\left(\theta\right)=\frac{1}{2}\lambda\cos\theta\sin\theta=\frac{1}{4}\lambda\sin\left(2\theta\right). \tag{S8}$$

In the presence of a potential energy barrier, the velocity component normal to the surface must exceed the escape velocity, i.e. $(E+E_0)\cos^2\theta>E_0$, where E_0 is the escape threshold. Therefore, the escape probability can be written as

$$P\left(\theta,E;E_0\right)=\begin{cases}\frac{1}{2}\lambda\cos\theta\sin\theta & (E+E_0)\cos^2\theta>E_0\\ 0 & \text{otherwise}\end{cases}. \tag{S9}$$

The fraction of electrons transmitted at the surface is thus given by

$$P\left(E;E_0\right)=\frac{\lambda}{4\pi}\int_{0}^{2\pi}\int_{0}^{\alpha(E)}\cos\theta\sin\theta\,\mathrm{d}\theta\,\mathrm{d}\phi, \tag{S10}$$

where $\alpha(E) = \arccos\left(\sqrt{\frac{E_0}{E+E0}}\right)$. Integrating over θ and ϕ gives

$$P(E; E_0) = \frac{\lambda}{4} \cdot \frac{E}{E + E_0}. \tag{S11}$$

The transmittance at the surface $T(E)$ is given by the escape probability relative to the escape probability when $E_0 = 0$:

$$T(E) = \frac{E}{E + E_0}. \tag{S12}$$

However, it is unlikely for the depth distribution to be uniform, as electrons near the surface have a higher probability of escaping. Therefore, we performed a Monte Carlo simulation of this simplified model by propagating 10^5 electrons with starting depths between 0.1 and 100 nm in 0.1 nm steps and limiting their trajectories to a maximum of 1 000 steps. If the depth of an electron became greater than 100 nm, the electron was specularly reflected to avoid unwanted depletion of electrons in the model. The trajectories of electrons that travelled through the surface were terminated and the angle of incidence on the surface determined. Our results show a distribution of angles with a peak below $\pi/4$ (Figure S5a). In an equivalent smaller simulation in which the number of electrons was 10^4 and trajectories were limited to 100 steps, we extracted the distribution of the depth from the surface of all electrons at each step and found it to be well described by a cumulative exponential distribution (Figure S5b).

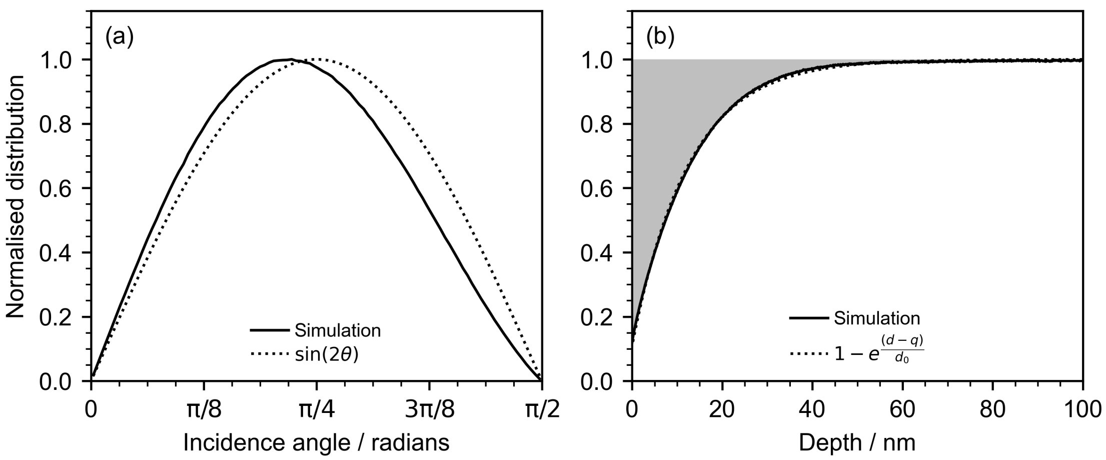

Figure S5: (a) Distribution of angles of incidence with the surface for electrons propagating under a semi-infinite flat surface. (b) Distribution of the depth of all electrons at all steps of the simulation (solid) fit with a shifted cumulative exponential distribution (dotted). The shaded area indicates the difference between the simulation and a uniform distribution. The distributions were obtained by performing random walks for electrons initialised at depths between 0.1 and 100 nm in 0.1 nm steps. Step lengths were sampled from an exponential distribution with a mean of 2.9 nm and electron moved in a random direction. Trajectories were stopped after the electrons reached the surface. In (a) 10^5 electrons were propagated and trajectories were limited to 10^3 steps. In (b) 10^4 electrons were propagated and trajectories were limited to 10^2 steps.

To test the effect of the distribution of depths, we determined the distribution of the angles of incidence using Monte Carlo integration and different depths profiles as follows. We sampled a depth between 0 and 50 nm using a uniform distibution, an exponential distribution, or a cumulative exponential distribution. We then sampled a step length from an exponential distribution and moved the electron in a random isotropic direction. If, after moving, the electron was outside the surface, we measured the angle with the normal to the surface; otherwise, it was discarded. We repeated this process 10^8 times and obtained a distribution of incidence angles for the three depth profiles (Figure S6). Our results show that the presence of a non-uniform depth distribution of electrons inside the jet causes the distribution of incidence angles to deviate from a $\sin(2\theta)$ distribution and that the depletion of electrons near the surface shifts the maximum of the distribution to lower incidence angles.

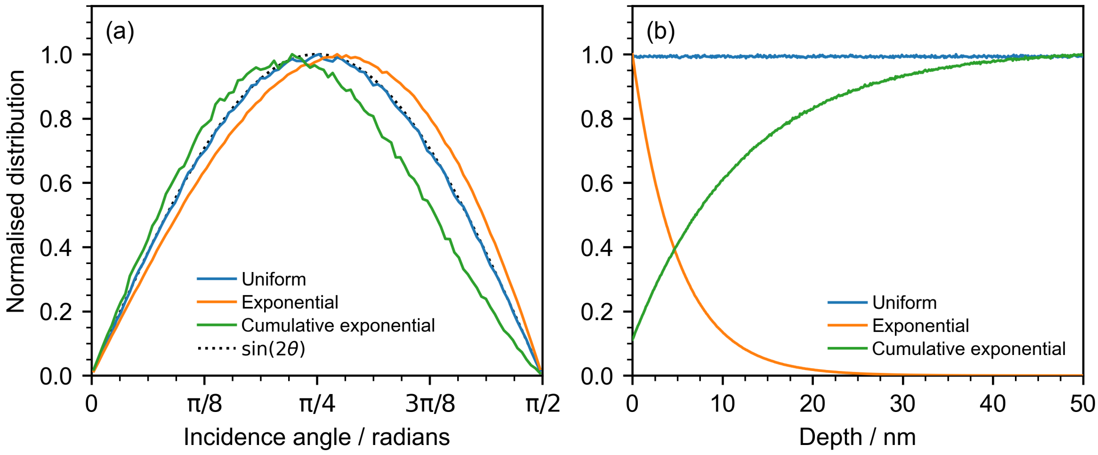

Figure S6: (a) Distribution of incidence angles obtained using Monte Carlo integration and a uniform distribution, an exponential distribution or a cumulative exponential distribution of depths (b) Distributions of depth employed to determine the angular distributions in (a) normalised to their maximum.

Inspired by the results in Figure S5 we used a cumulative exponential distribution with mean d_0 to scale d before integrating over d,

$$I(\theta) = \frac{1}{2}\sin\theta \int_0^{d_{\max}} \mathrm{e}^{-\frac{d}{\lambda\cos\theta}} \left(1 - \mathrm{e}^{-\frac{d}{d_0}}\right) \mathrm{d}d. \tag{S13}$$

Assuming $d_{\max} \gg \lambda$ and $d_{\max} \gg d_0$ and integrating over d gives

$$I(\theta) = \frac{\lambda^2\cos^2\theta\sin\theta}{2(\lambda\cos\theta + d_0)}. \tag{S14}$$

The escape probability is then given by

$$P(E; E_0) = \int_0^{\alpha(E)} \frac{\lambda^2\cos^2\theta\sin\theta}{2(\lambda\cos\theta + d_0)} \mathrm{d}\theta. \tag{S15}$$

Integrating over θ gives

$$\begin{aligned} P(E; E_0) =& \frac{\lambda}{4}\cdot\frac{E}{E+E_0} + \frac{d_0}{2}\left(\sqrt{\frac{E_0}{E+E_0}} - 1\right) \\ & - \frac{d_0^2}{2\lambda}\ln\left(\lambda\sqrt{\frac{E_0}{E+E_0}} + d_0\right) + \frac{d_0^2}{2\lambda}\ln(\lambda + d_0)\,. \end{aligned} \tag{S16}$$

The transmittance is given by

$$T(E; E_0) = \frac{P(E; E_0)}{P(E; 0)} \tag{S17}$$

Figure S7 shows a comparison between the transmittance determined using eqs S7, S12 and S17 (full lines) compared to the transmittance obtained from the Monte Carlo simulations shown in Figure 3c in the main text (shaded lines). eq S17 was fit to the data in Figure 3c (main text) to obtain values for λ and d_0.

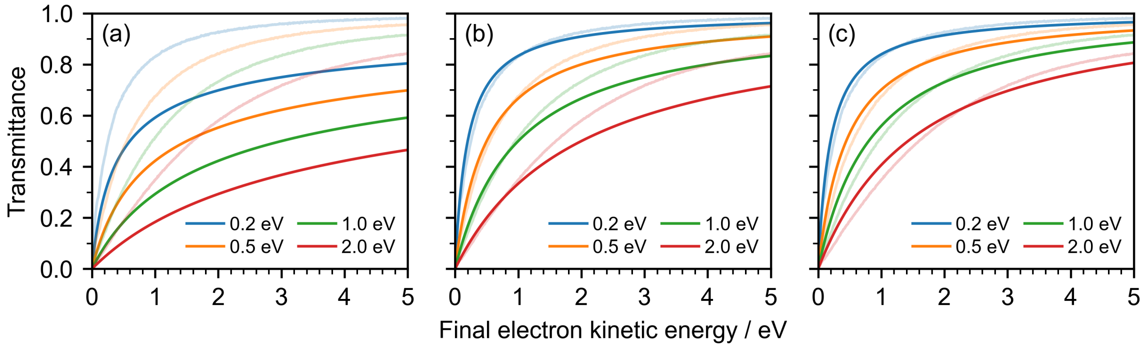

Figure S7: Transmittance determined using (a) eq S7, (b) eq S12 and (c) eq S17 (full lines). Shaded lines indicate the transmittance obtained from the Monte Carlo simulations shown in the main text using method **B** and cross-section set **III**. Eq S17 was fit to the shaded lines to obtain values for λ and d_0.

S6. Sensitivity analysis

In order to verify the choice of parameters employed in our work, we tested the effect of changing several input parameters on the result of our code. For the sensitivity study described in this section, cross-section set **III** and method **B** (see main text) were employed in the Monte Carlo simulations.

S6.1. Number of starting electrons

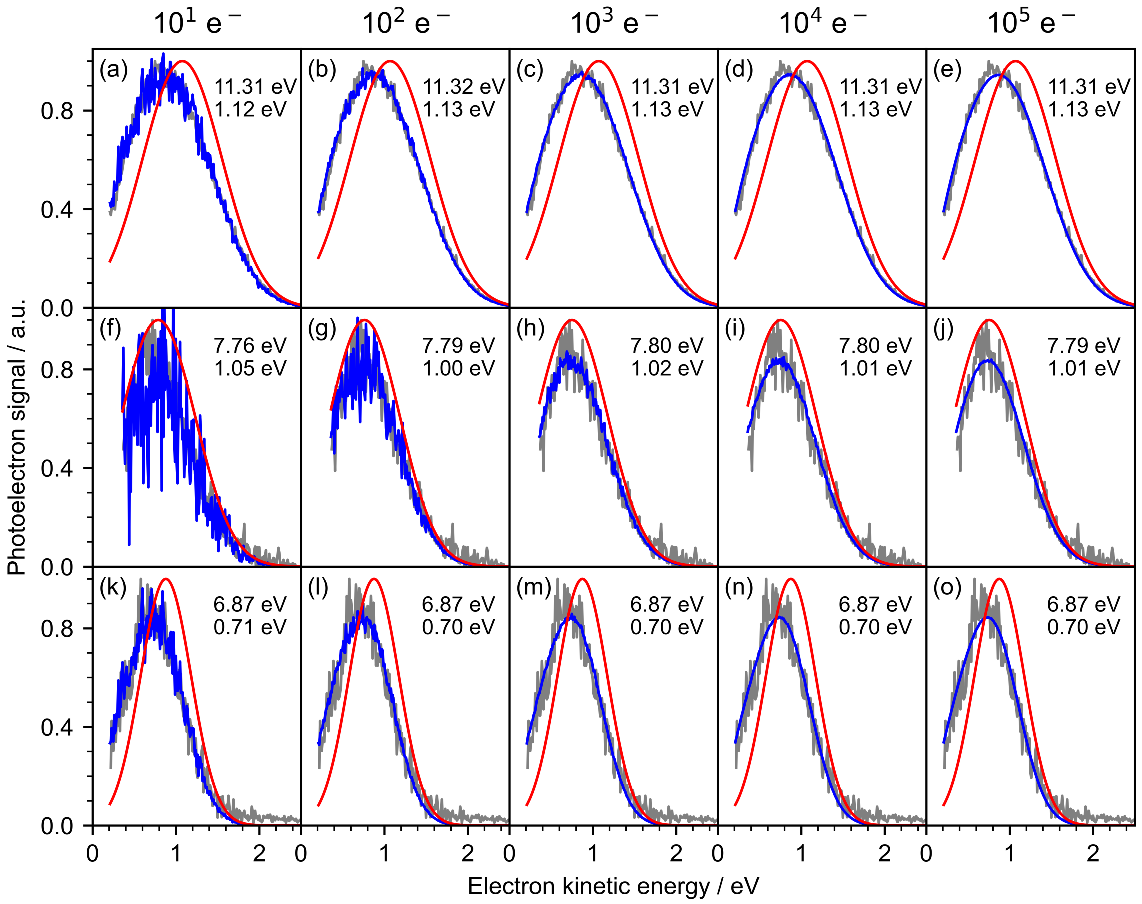

Figure S8: Effect of the number of starting electrons at each depth on the retrieval of the spectra of (a-e) water, (f-g) phenol and (k-o) phenolate. Gray lines are the experimental data, blue lines are fits to the data, red lines are retrieved spectra, and numbers in each panel are the retrieved eBEs (top) and FWHMs (bottom). Experimental spectra reproduced from ref 2. Copyright 2022 American Chemical Society.

Figure S8 shows the retrieval of the photoelectron spectra of water, phenol and phenolate as a function of the number of starting electrons employed at each depth and starting eKE in the Monte Carlo simulation. Our choice to employ 10^4 electrons per E_i and d was driven by the fact that the retrieved photoelectron spectra for bulk water and surface bound organic solutes were consistent with those retrieved using a larger number of electrons. Furthermore, the fits (blue) have an acceptable signal-to-noise ratio when 10^4 electrons are used.

S6.2. Simulation depth and step size

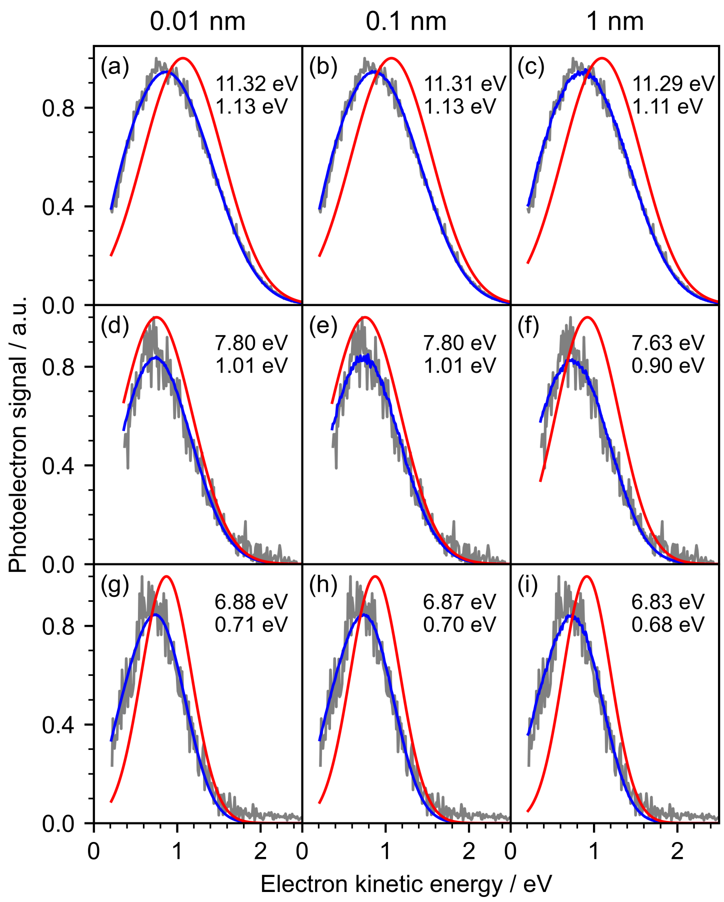

Figure S9: Effect of the step size used in the sampling of d on the retrieval of the photoelectron spectra of (a-c) water, (d-f) phenol and (g-i) phenolate. Electrons were initialised at depths between 0 and 50 nm. Gray lines are the experimental data, blue lines are fits to the data, red lines are retrieved spectra, and numbers in each panel are the retrieved eBEs (top) and FWHMs (bottom). Experimental spectra reproduced from ref 2. Copyright 2022 American Chemical Society.

Figure S9 shows the effect of the step size used when sampling the depths from the surface of the jet on the retrieval of the photoelectron spectra of water, phenol and phenolate. Using a step size of 0.1 nm or smaller results in consistent results. A step size of 1 nm shifts the eBE of water and phenolate by only a few tens of meV because most of the signal comes for the bulk. The eBE of phenol is shifted by 0.2 eV as the step size is too large to properly account for the enhanced surface contribution.

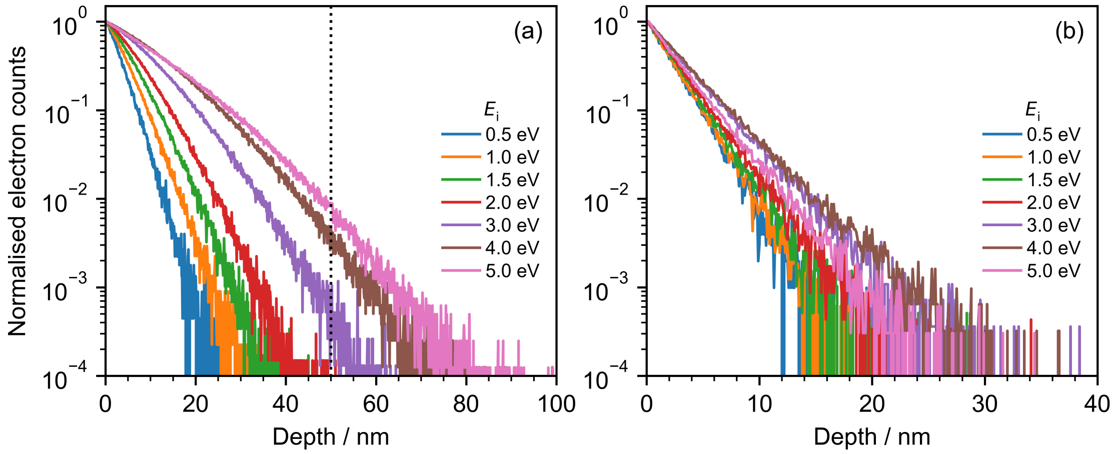

Figure S10: (a) Distribution of electrons that successfully escape the jet as a function of depth for different initial kinetic energies. (b) Distribution of electrons that maintain their original eKE and successfully escape the jet as a function of depth for different initial kinetic energies. All distributions were normalised to the number of electrons that escape at a depth of 0.1 nm. Depths between 0.1 and 100 nm and a step size of 0.1 nm were employed. This simulation was run using method **B** with an escape threshold of 1.0 eV and cross-section set **III**.

Figure S10a shows the distribution of electrons that escape the jet as a function of depth for depths between 0.1 and 100 nm and several starting eKEs. Most electrons originate from within a few tens of nm below the surface. We employed a maximum depth of 50 nm in our simulation as the contribution of electrons from below this depth is negligible. Figure S10b shows the distribution of electrons that escape the jet and maintain their original eKE as a function of depth. The effective attenuation length ranges from 2.1 to 3.1 nm for 0.5 to 5 eV electrons.

S6.3. Radius of the jet

Due to evaporation, the size of the jet at the interaction region is likely smaller than the size of the fused silica nozzle. Therefore, we tested the effect of jet radii between 0.5 and 25 µm on the retrieval of the photoelectron spectra of water, phenol and phenolate and found virtually no difference in the retrieved spectra (Figure S11).

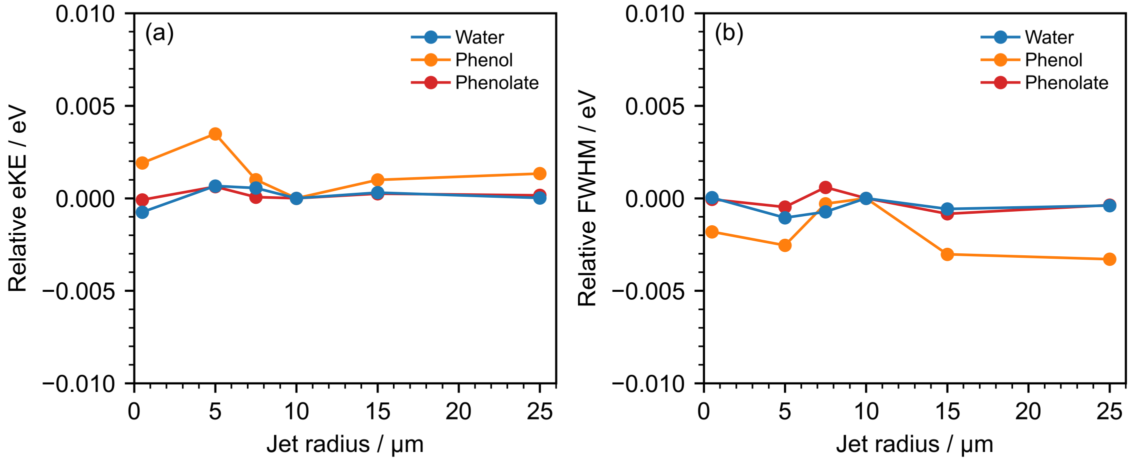

Figure S11: Effect of the radius of the jet on the retrieval of the photoelectron spectra of water, phenol and phenolate. (a) Change in the central eKE relative to the eKE retrieved with a radius of 10 µm. (b) Change in the FWHM relative to the FWHM retrieved with a radius of 10 µm. Simulations were run using method **B** with an escape threshold of 1.0 eV and cross-section set **III**.

S6.4. Relative contribution of elastic and inelastic cross sections

We tested the effect of the relative contribution of the elastic and total inelastic cross sections on the retrieval of the photoelectron spectra of water, phenol and phenolate, by multiplying their ratio by a scaling factor, while keeping their sum constant (Figure S12). For Gaussians with centres below 1.5 eV, as is the case for the UV photoelectron spectra presented in this work, there is very little difference ($<$ 0.03 eV) as the escape threshold has a large effect. For Gaussians centred at higher eKEs, increasing the relative contribution of the elastic cross sections has a noticeable effect on the distorted distribution (Figure S13).

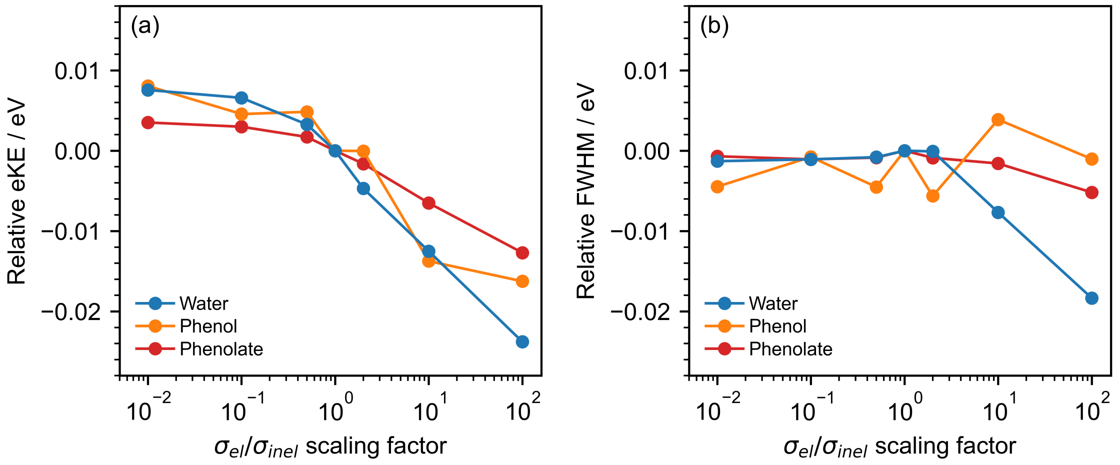

Figure S12: Effect of the relative contribution of the total inelastic and elastic cross sections on the retrieval of the photoelectron spectra of water, phenol and phenolate. The ratio of elastic to total inelastic cross sections was multiplied by a scaling factor, while their sum was kept constant. (a) Change in the central eKE relative to the eKE retrieved with no scaling factor applied. (b) Change in the FWHM relative to the FWHM retrieved with no scaling factor applied. Simulations were run using method **B** with an escape threshold of 1.0 eV and cross-section set **III**. Concentration profiles with Gaussian-offset ratios of 20 and 1 were employed for phenol and phenolate, respectively.

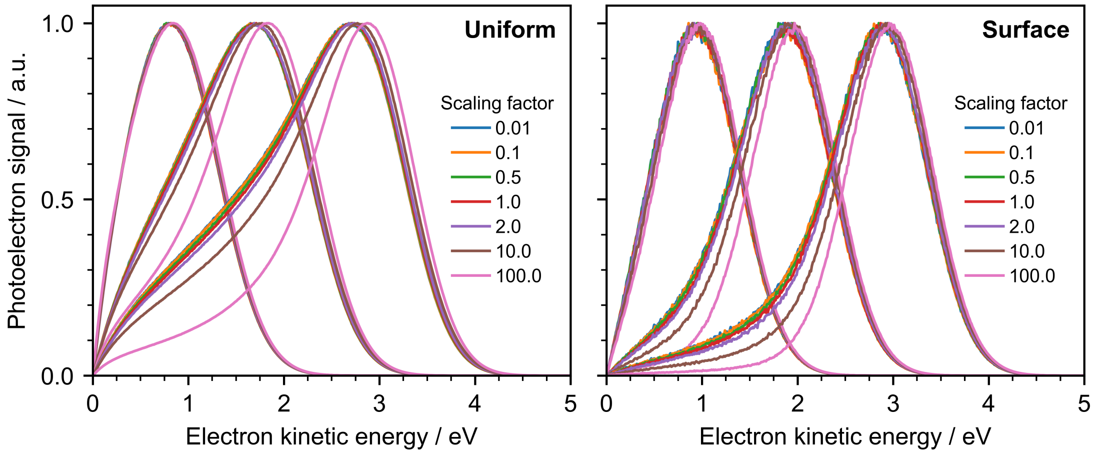

Figure S13: Effect of the relative contribution of the total inelastic and elastic cross sections on the distortion of Gaussians centred at 1, 2 and 3 eV. The ratio of elastic to total inelastic cross sections was multiplied by a scaling factor, while their sum was kept constant (a) A uniform concentration profile was employed. (b) A concentration profile with a Gaussian to offset ratio of 20 was employed. Simulations were run using method **B** with an escape threshold of 1.0 eV and cross-section set **III**.

S7. Uncertainties in the spectral retrieval

The accuracy of our spectral retrieval depends on the uncertainties associated with our experimental data ($\pm$ 0.07)[2] and the uncertainties in our model. Similarly to our previous work,[2] we assume that the energy-loss parameters obtained from electron-scattering experiments in amorphous ice have an accuracy of at least 0.01 eV. We expect the uncertainty in the magnitude of the cross sections to be less than 0.03 eV, based on the results shown in S6 and the overall uncertainty associated with rounding, the number of electrons, the depth step size, the maximum probed depth, the radius of the jet employed in our simulations to be less than 0.02 eV. Therefore, we expect the errors associated with the retrieval of UV LJ-PES spectra to be $\pm$ 0.04 and quote the retrieved VIE and VDEs of water, phenol and phenolate to an accuracy of $\pm$ 0.09 eV.

S8. Solvent-only background photoelectron spectra

Figure S14 shows the solvent-only spectra of 1.75 mM NaF(aq) and 2.0 mM NaOH(aq). These spectra were fit with 'bulk' Gaussians which were subtracted from the spectra in Figures 7a,b in the main text to give the spectra in Figures 7c,d.

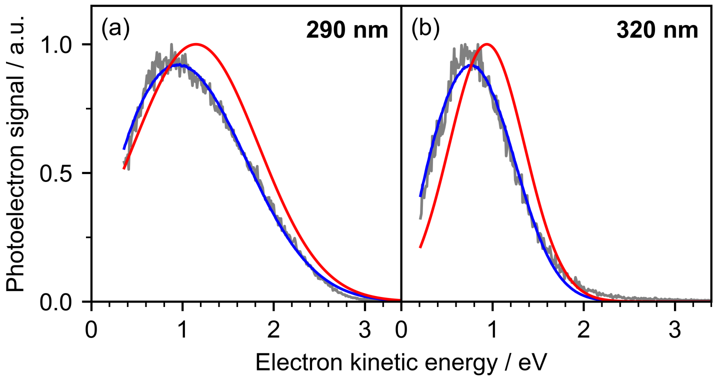

Figure S14: Photoelectron spectra of (a) 1.75 mM NaF(aq) recorded at 290 nm and (b) 2.0 mM NaOH(aq) recorded at 320 nm fit with 'bulk' Gaussians. Grey lines are the experimental spectra, blue lines are fits to the data, red lines are retrieved spectra. All spectral retrievals employed cross-section set **III**, and method **B** with an escape threshold of 1 eV.

References

[1] Michaud, M.; Wen, A.; Sanche, L. Cross sections for low-energy (1-100 eV) electron elastic and inelastic scattering in amorphous ice. *Radiation Research* **2003**, *159*, 3–22.

[2] Scholz, M. S.; Fortune, W. G.; Tau, O.; Fielding, H. H. Accurate Vertical Ionization Energy of Water and Retrieval of True Ultraviolet Photoelectron Spectra of Aqueous Solutions. *The Journal of Physical Chemistry Letters* **2022**, *13*, 6889–6895.

[3] Gadeyne, T.; Zhang, P.; Schild, A.; Wörner, H. J. Low-energy electron distributions from the photoionization of liquid water: a sensitive test of electron mean free paths. *Chemical Science* **2022**, *13*, 1675–1692.

[4] Eastman, P.; Swails, J.; Chodera, J. D.; McGibbon, R. T.; Zhao, Y.; Beauchamp, K. A.; Wang, L.-P.; Simmonett, A. C.; Harrigan, M. P.; Stern, C. D.; Wiewiora, R. P.; Brooks, B. R.; Pande, V. S. OpenMM 7: Rapid development of high performance algorithms for molecular dynamics. *PLOS Computational Biology* **2017**, *13*, e1005659.

[5] Malde, A. K.; Zuo, L.; Breeze, M.; Stroet, M.; Poger, D.; Nair, P. C.; Oostenbrink, C.; Mark, A. E. An Automated Force Field Topology Builder (ATB) and Repository: Version 1.0. *Journal of Chemical Theory and Computation* **2011**, *7*, 4026–4037.

[6] Stroet, M.; Caron, B.; Visscher, K. M.; Geerke, D. P.; Malde, A. K.; Mark, A. E. Automated Topology Builder Version 3.0: Prediction of Solvation Free Enthalpies in Water and Hexane. *Journal of Chemical Theory and Computation* **2018**, *14*, 5834–5845.

[7] Mobley, D. L.; Bannan, C. C.; Rizzi, A.; Bayly, C. I.; Chodera, J. D.; Lim, V. T.; Lim, N. M.; Beauchamp, K. A.; Slochower, D. R.; Shirts, M. R.; Gilson, M. K.; Eastman, P. K. Escaping Atom Types in Force Fields Using Direct Chemical Perception. *Journal of Chemical Theory and Computation* **2018**, *14*, 6076–6092.

[8] Jorgensen, W. L.; Chandrasekhar, J.; Madura, J. D.; Impey, R. W.; Klein, M. L. Comparison of simple potential functions for simulating liquid water. *The Journal of Chemical Physics* **1983**, *79*, 926–935.

[9] Zhang, Z.; Liu, X.; Yan, K.; Tuckerman, M. E.; Liu, J. Unified Efficient Thermostat Scheme for the Canonical Ensemble with Holonomic or Isokinetic Constraints via Molecular Dynamics. *The Journal of Physical Chemistry A* **2019**, *123*, 6056–6079.

[10] Winter, B.; Weber, R.; Widdra, W.; Dittmar, M.; Faubel, M.; Hertel, I. V. Full Valence Band Photoemission from Liquid Water Using EUV Synchrotron Radiation. *Journal of Physical Chemistry A* **2004**, *108*, 2625–2632.

[11] Nishizawa, K.; Kurahashi, N.; Sekiguchi, K.; Mizuno, T.; Ogi, Y.; Horio, T.; Oura, M.; Kosugi, N.; Suzuki, T. High-resolution soft X-ray photoelectron spectroscopy of liquid water. *Physical Chemistry Chemical Physics* **2011**, *13*, 413–417.

[12] Kurahashi, N.; Karashima, S.; Tang, Y.; Horio, T.; Abulimiti, B.; Suzuki, Y.-I.; Ogi, Y.; Oura, M.; Suzuki, T. Photoelectron spectroscopy of aqueous solutions: Streaming potentials of NaX (X = Cl, Br, and I) solutions and electron binding energies of liquid water and X−. *The Journal of Chemical Physics* **2014**, *140*, 174506.

[13] Perry, C. F.; Zhang, P.; Nunes, F. B.; Jordan, I.; Von Conta, A.; Wörner, H. J. Ionization Energy of Liquid Water Revisited. *Journal of Physical Chemistry Letters* **2020**, *11*, 1789–1794.

[14] Thürmer, S.; Shinno, T.; Suzuki, T. Valence Photoelectron Spectra of Liquid Methanol and Ethanol Measured Using He II Radiation. *Journal of Physical Chemistry A* **2021**, *125*, 2492–2503.

[15] Thürmer, S.; Malerz, S.; Trinter, F.; Hergenhahn, U.; Lee, C.; Neumark, D. M.; Meijer, G.; Winter, B.; Wilkinson, I. Accurate vertical ionization energy and work function determinations of liquid water and aqueous solutions. *Chemical Science* **2021**, *12*, 10558–10582.

[16] Goulet, T.; Jay-Gerin, J.-P.; Patau, J.-P. Monte carlo simulations of low-energy (<10eV) electron transmission and reflection experiments: application to solid xenon. *Journal of Electron Spectroscopy and Related Phenomena* **1987**, *43*, 17–35.

[17] Nishitani, J.; Yamamoto, Y.-i.; West, C. W.; Karashima, S.; Suzuki, T. Binding energy of solvated electrons and retrieval of true UV photoelectron spectra of liquids. *Science Advances* **2019**, *5*, eaaw6896.

[18] Yamamoto, Y.-I.; Suzuki, T. Distortion Correction of Low-Energy Photoelectron Spectra of Liquids Using Spectroscopic Data for Solvated Electrons. *The Journal of Physical Chemistry A* **2023**, *127*, 2440–2452.
